# Neuropsychiatric aspects of concussion: acute and chronic sequelae

**DOI:** 10.2217/cnc-2016-0018

**Published:** 2017-02-02

**Authors:** Vani Rao, Arshiya Syeda, Durga Roy, Matthew E Peters, Sandeep Vaishnavi

**Affiliations:** 1 Department Of Psychiatry, Johns Hopkins University & School of Medicine, 5300 Alpha Commons Drive, Baltimore, MD, USA; 2 The Preston Robert Tisch Brain Tumor Center, Duke Medicine; Department of Psychiatry & Behavioral Sciences & Community & Family Medicine, Duke University Medical Center, Durham, NC, USA; The Neuropsychiatric Clinic at Carolina Partners, Raleigh, NC, USA

**Keywords:** chronic traumatic encephalopathy, concussion, neuropsychiatric symptoms, traumatic brain injury

## Abstract

Concussion – also known as mild traumatic brain injury – is a transient disturbance of neurological function resulting from traumatic forces imparted to the brain that often produce cognitive, behavioral and systemic symptoms. In this review of the literature, we discuss the pathophysiology of both acute and chronic neuropsychiatric sequelae of concussions, followed by a brief overview of evaluation and management of these sequelae.

Traumatic brain injury (TBI) is a growing public health problem. In 2010, suspected TBI accounted for approximately 2.5 million emergency department visits in the USA [1]. About 70–80% of all TBIs are mild (mTBI) and the term concussion is often used interchangeably with mTBI. mTBI is defined as a complex biological process affecting the brain induced by traumatic biomechanical forces to the head. The United States Department of Defense diagnostic criteria for mTBI include: loss of consciousness for 0–30 min, alteration of consciousness/mental state from a moment up to 24 h, post-traumatic amnesia for up to 24 h, Glasgow coma scale of 13–15 and normal structural imaging scan. In this review article, henceforth, we will use the term concussion.

Neuropsychiatric symptoms (NPS) are common after concussion and are typically transient, often resolving in 7–10 days. In some cases, NPS persist for months to years and cause cognitive impairments, emotional disturbances and/or behavioral problems [2]. Presence of persistent NPS requires a comprehensive medical and psychiatric work-up because they may be caused by factors other than concussion [3,4]. The overarching goal of this literature review is to discuss the acute and chronic NPS associated with concussion. The acute NPS include postconcussion syndrome and the second-impact syndrome (SIS). Chronic NPS include emotional/behavioral disturbances and cognitive deficits. We will discuss each in turn, then provide a brief overview of evaluation and management, concluding with directions for the future.

## Acute neuropsychiatric symptoms/syndromes

### Postconcussion syndrome

Soon after a concussion, the majority of people experience one or more distressing symptoms that may be grouped into three categories: emotional (e.g., depression, anxiety, anger, irritability), cognitive (e.g., inattention, decreased information processing speed, dysexecutive function) and physical (e.g., headache, nausea, vertigo, increased sensitivity to noise or light). These symptoms, known as postconcussive symptoms, usually persist from a few days to weeks after injury. Persistence of symptoms beyond this generally accepted time period has been known by various names such as postconcussion syndrome, persistent postconcussion symptoms [5] and delayed symptom resolution [6]. Confusion exists in the literature regarding the time duration for the resolution of acute symptoms, with estimates ranging from 1 month to 6 months.

Postconcussive symptoms are subjective and nonspecific. They can be encountered in nonbrain injuries, general medical illnesses and other neuropsychiatric disorders, thus increasing the risk of misdiagnosis [7,8]. The International Collaboration on Mild Traumatic Brain Injury Prognosis (ICoMP) [9] posits that symptoms experienced subsequent to concussions are nonspecific (i.e., typical reactions to the injury stress) and clinicians should be cautious about attributing common postinjury symptoms to a concussion. There is evidence that for most people with concussion and normal structural brain scan the prognosis is good, with spontaneous recovery and no long-term adverse outcomes [10–12]. However, a small percentage of patients, ranging from 5 to 15%, continue to have persistent mood and cognitive symptoms [4,13,14]. Maruta *et al*. [15] studied the association of persistent symptoms in 33 postconcussive patients, noting that compared with healthy controls, there were no isolated deficits in specific cognitive domains, but there was reduced information-processing efficiency and fatigability, suggesting a possible alteration of the attentional network under increased cognitive stress, even when controlling for comorbid psychiatric symptoms.

Even though there is no conclusive evidence on various factors associated with the persistence of postconcussive symptoms, several have been hypothesized. Examples include preinjury medical or psychosocial factors, difficulty adjusting to the effects of injury [16], post-TBI development of psychiatric illness (e.g., major depressive disorder or post-traumatic stress disorder) [17,18], alcohol or substance abuse, embellishing symptoms for personal gain [19] and repeated TBIs. In a study of 586 individuals with TBI (82% of which were concussions) [20], it was noted that 23% had a previous TBI. In comparing individuals with previous TBI to those without previous TBI, the former were noted to report more postconcussive symptoms and poorer satisfaction with life. Other factors that should be considered when making a diagnosis of postconcussive syndrome include premorbid intelligence, the patient's illness perception, effort placed by the patient on objective tests, ongoing litigation and possible malingering [21]. The ICoMP emphasizes that psychosocial factors and co-morbidities in patients with persistent postconcussion symptoms need to be addressed and modified. They strongly recommend refraining from additional diagnostic testing that have the potential to cause unnecessary anxiety and worsen the postconcussive symptoms [22].

With regards to the diagnosis of postconcussion syndrome, the Diagnostic and Statistical Manual of Mental Disorders (DSM)-IV-R first used the term ‘postconcussional disorder’ or ‘postconcussional syndrome’ and placed it under the criteria domain of Cognitive Disorder Not Otherwise Specified [23]. It was presented as a research criteria that required further study and the criteria differed from the ICD-9-CM criteria for postconcussive syndrome. Secondary to this confusion and its lack of usefulness in research and in clinical practice, DSM-5 has eliminated this term and the syndrome is now subsumed under the heading of neurocognitive disorder due to TBI. Neurocognitive disorder is further divided as mild (if the cognitive deficits do not interfere with independence in everyday activities) and major (if the cognitive deficits interfere with independence in everyday activities). As Wortzel and Arciniegas [23] have pointed out, the approach to diagnosing and managing NPS associated with TBI is significantly improved in DSM-5 as it offers a clear definition of TBI, criteria for rating the severity, provides the option of specifying with or without behavioral disturbances, describes the expected course of recovery associated with the different severities of TBI and recommends clinicians to consider explanations other than TBI when recovery is atypical.

### Second-impact syndrome

Schneider *et al*. [24] first described the clinical picture of two young athletes who experienced postconcussive symptoms after an initial injury and died after a relatively minor second head injury. The term SIS was coined by Saunders and Harbaugh in 1984 [25]. Autopsy of one of the cases revealed extensive cerebral edema, but absence of a space-occupying lesion [26].

Since then, SIS has been described as a condition that occurs after sustaining a second TBI before symptoms from the first TBI have resolved. The second impact can occur days to weeks after the first, and can be relatively mild compared with the first. Typically, after the first trauma, the person experiences a number of physical and cognitive symptoms, such as headache, dizziness, confusion and short-term memory problems, but returns to play before complete resolution of these symptoms. Within seconds to minutes of the second injury, the person collapses secondary to severe cerebral edema and brain herniation [27].

The pathophysiology of SIS is slightly controversial. SIS has been linked to abrupt trauma-induced cerebral vascular dysregulation and release of catecholamines followed by a rapid increase in intracranial blood volume and cerebral edema, in turn increasing intracranial pressure and causing subsequent herniation and brainstem compression [28–30]. The usual time period from the second impact to brainstem failure is rapid, taking 2–5 min. Once brain herniation and brainstem compromise occur, ocular involvement and respiratory failure precipitously ensue [31]. An acute, small subdural hematoma has been reported in most cases on neuroimaging or at autopsy [32]. Some investigators have proposed that subdural hematoma is the major cause of brain swelling, but others [27,33] have argued that both cerebral vascular dysregulation and subdural hematoma are due to acceleration–deceleration forces. It has been hypothesized that the cerebral vascular dysregulation is caused by reinjury to neurons within a vulnerable period following the previous injury [34,35]. Brain imaging findings in ten cases of SIS [31] included the following: maximal thickness of the subdural hematoma was <0.5 cm, heterogeneity of the subdural hematoma, complete effacement of the basal cisterns and cerebral sulci, morphological distortion of the brainstem due to uncal and diencephalic herniation, absence of intra-axial hemorrhage such as contusions or diffuse axonal injury, preservation of the gray-white matter differentiation within the cerebral hemispheres on admission CT, hemispheric asymmetry and multifocal ischemic infarction on follow-up imaging studies in survivors.

In summary, the incidence and pathogenesis of SIS are unclear. In particular, it is unclear why the catastrophic brain response requires only a mild second TBI event. We also need more research on the risk factors and prevention of SIS. Still, one strong recommendation from the current case reports is that premature return to play in some athletes who remain symptomatic after a first concussion should be strongly discouraged. At present, a unique presentation scheme of SIS to support a standardized WHO case definition is lacking. A recent prospective study of 69 athletes by Elbin *et al*. [36] revealed that athletes who were not immediately removed from play after a sports-related concussion compared with those who were immediately removed took longer to recover and were 8.80-times more likely to demonstrate protracted recovery as defined by time to return to full play and neurocognitive performance. Future studies are needed to better understand and define at-risk populations, diagnostic signs and symptoms and the multisystem consequences of SIS [37].

## Chronic neuropsychiatric symptoms/syndromes

### Emotional/behavioral disturbances

Several studies have noted that the burden of prior TBI is positively correlated with the degree of emotional distress and rate of substance abuse. In a study of 161 military personnel referred for evaluation and treatment of suspected head injury, Bryan [38] noted a direct relationship between symptoms of depression or post-traumatic stress disorder, and number of previous TBI events. Dams-O'Connor *et al*. [20] found worse injury outcomes in individuals with previous TBI (82% of the study sample had concussion, 5% moderate and 12% severe) in several domains: emotional (more mood symptoms), behavioral (greater substance abuse), cognitive (slower processing speed, poorer verbal learning) and overall quality of life. A survey of 2552 retired professional football players [39] using a general health questionnaire and the SF-36 (a self-reported survey measure of health status) found that players who reported three or more previous concussions were three-times more likely to be diagnosed with depression, whereas those with one or two concussions had 1.5-times higher risk. The major limitation of this study was the use of a questionnaire with self-report of symptoms and diagnosis. However, the analysis was comprehensive and controlled for several variables: age, number of years since retirement, number of years played, physical component score on the SF-36 and medical co-morbidities (e.g., osteoarthritis, coronary heart disease, stroke, cancer and diabetes).

Insomnia, a common problem after TBI, may be further aggravated with additional TBI events [38,40]. In a study of 150 male military patients (severity of TBI not described in this study), it was noted that 50% of those with multiple TBIs reported insomnia compared with only 20% of subjects with single TBI and 6% of subjects with no TBI. In addition, the severity of insomnia also increased significantly with higher TBI burden after controlling for depression, post-traumatic stress disorder and concussion symptom severity.

Corrigan *et al*., in a study of 257 outpatients with lifetime histories of both TBI (at least 51% with mild TBI) and substance use disorder [43] noted that individuals with severe injury in adult age, mild injury in adolescence or multiple mild injuries had greater working memory impairments and greater severity of alcohol use disorders.

The role of mild TBI in suicide is controversial. Stanley *et al*., [41] in a study of a sample of military personnel, noted that suicide in concussion is often associated with increased anger and depression. Wortzel *et al*. [42] correctly pointed out that, “the quality of evidence regarding a relationship between concussion and suicide is very low”. More studies are needed in order to clarify interactions between concussions, psychiatric disorders and suicide, and use this understanding to improve clinical practices.

### Cognitive deficits

There are few studies that have examined the impact of concussions on neuropsychological testing. Wall *et al*. [43] described 698 jockeys who experienced repeat concussions and found an association with poor performance on neuropsychological testing (i.e., impairments in response inhibition and divided attention). Guskiewicz *et al*. [39] investigated the link between repeated concussions and the development of late-life cognitive impairment in retired professional football players. Health questionnaires were mailed to 3683 retired National Football League players. Responses were obtained from 2552 with a mean age of 53.8 years and average professional football playing career of 6.6 years. A subset of 758 completed a second questionnaire evaluating cognitive symptoms. Analyses revealed that players with three or more concussions had a fivefold greater prevalence of mild cognitive impairment and a threefold greater prevalence of significant memory problems compared with players without a history of concussions. There was no statistically significant association between recurrent concussions and Alzheimer's disease, although the authors noted an earlier onset of Alzheimer's disease in these players compared with the general male American population.

Belanger *et al*. [10] conducted a meta-analysis of the relevant literature to determine the impact of multiple concussions on cognition. The analysis was based on eight studies, all of which were conducted on athletes. Their total sample included 614 cases of multiple concussions and 926 control cases with single concussions. They found that the overall effect of multiple concussions was not significant. However, multiple self-reported concussions were associated with poorer performance on measures of delayed memory and executive functioning. Also, when they looked at cognitive domains as a moderator, both executive function and delayed memory were associated with multiple self-reported concussions. The authors concluded, “given the exploratory nature of the moderator analyses, further prospective studies need to be done.” Gronwall *et al*. [44] also reported that, compared with individuals who had suffered one concussion, those who suffered two concussions performed worse on a test of processing speed, a pattern suggesting that the effects of concussion are cumulative with each event, a pattern hypothesized to result from progressive destruction of neurons and depletion of the cognitive reserve.

The relationship between TBI and dementia is interesting. According to the report by the Institute of Medicine [45], “there is sufficient evidence of an association between moderate and severe TBI and dementia … limited/suggestive evidence of an association between mild TBI (with loss of consciousness) and dementia … (and) inadequate/insufficient evidence to determine whether an association exists between mild TBI (without loss of consciousness) and dementia.” Shively *et al*. [46] in a review article on dementia and TBI conclude that, “the best data indicate that moderate and severe TBIs increase risk of dementia between two- and four-fold. It is less clear whether mild TBIs such as brief concussions result in increased dementia risk, in part because mild head injuries are often not well documented and retrospective studies have recall bias.”

However, not all agree on the increased risk of neuropsychiatric symptoms after repeated concussion. Silverberg *et al*. [47] examined 105 participants 1 month after sustaining a concussion. Approximately half the sample had at least one previous concussion. Subgroups with 0, 1 or 2+ previous concussions did not differ in levels of current postconcussion symptom reporting on the British Columbia postconcussion Symptom Inventory.

Iverson *et al*. [48] studied 867 male high school and college amateur athletes who took the ImPACT computerized TBI test battery. Subjects were divided into three groups on the basis of number of previous concussions: 664 athletes had no previous concussions, 149 had one previous concussion and 54 had two previous concussions. Multivariate analysis of variance was conducted using verbal memory, visual memory, reaction time, processing speed and postconcussion symptom composite scores as dependent variables and group membership as the independent variable. There was no significant multivariate effect, nor was there any significant main effect for individual scores. There was no measurable effect of one or two previous concussions on athletes’ preseason neuropsychological test performance or symptom reporting.

In summary, even though there is a lot of discussion in the media about concussions and their sequelae, there are many gaps and discrepancies in the scientific literature. The major limitation is the absence of appropriately controlled longitudinal prospective studies exploring risk and protective factors associated with the development of chronic neuropsychiatric symptoms/syndromes in people with concussions. Other reasons include: lack of uniform methods to ascertain concussion, obtaining history of concussion retrospectively, a wide range in time duration from the time of concussion to clinical evaluation and inconsistencies in the use of psychiatric instruments (e.g., surveys, self-administered scales, single questions, structured evaluations). In addition, the literature on neuropsychiatric presentations varies by the types of outcome measures used, such as mood/anxiety symptoms, cognitive deficits, neurologic sequelae, functional deficits or a combination of these.

### Chronic traumatic encephalopathy

Chronic traumatic encephalopathy (CTE) has been recently proposed as a latent neurodegenerative condition in the context or history of playing in professional football and sustaining multiple concussions. It has been argued by some that this condition can be seen even after a single concussion [49–51]). Clinical symptoms include mood dysregulation (e.g., depression, lability, apathy), behavior (e.g., anger, aggression, impulsivity), cognition (e.g., short-term memory deficits, inattention, impaired problem solving, poor judgment) and sensorimotor (e.g., headache, Parkinsonism, gaze difficulties and incoordination). Pathological features include phosphorylated tau tangles in neurons and astrocytes in cortical and limbic regions of the brain, neuronal cell death and atrophy and 43-kDA TAR DNA-binding protein (TDP-43) positive inclusions inside neurons. Distribution of tau tangles predominates in perivascular regions and at the depths of cortical sulci [51].

The concept of CTE as a distinct neuropathological entity has been criticized [52,53]. The major concern is that the clinical-pathological data have been drawn from case reports and ‘samples of convenience’ with family members/friends requesting autopsy secondary to presence of NPS prior to the death of the person and a host of other reasons. The ICoMP note that there are no established clinical criteria for CTE and emphasize the uncertainty of the relationship between CTE and dementia, especially in cases involving concussions [54]. Other authors [52,53] emphasize that, until there are well-controlled epidemiological (prospective) studies on the risk factors associated with the development of neuropsychiatric symptoms in people with repeat concussions, it is too soon to reach a definitive conclusion about the neurodegenerative consequences of repeat concussions, even though there are signals indicating that repeated head injury is associated with a neurodegenerative process.

## Evaluation & management

### Evaluation

Currently there is no laboratory test that can be used to make the diagnosis of concussion, although there is a great move in academia and industry to develop concussion biomarkers and some of them, like S100B, BDNF and GFAP are increasingly promising [55]. The gold standard is clinical evaluation. Imaging studies and neuropsychological testing should be considered in people with history of more than one concussion, when history taking or clinical examination are inadequate (e.g., unreliable historian), or when symptoms persist.

**Figure 1.**
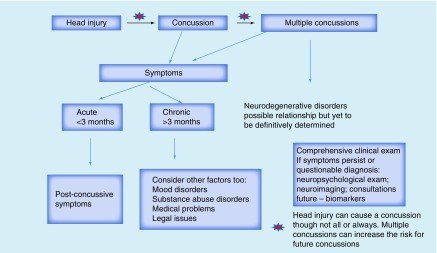
**Neuropsychiatric sequelae of concussions.**

The diagnosis of concussion and its neuropsychiatric consequences can be complex (see Figure 1). NPS can be transient or persistent and can remit and relapse. It is important to have a comprehensive formulation and be cautious about either attributing all symptoms to the injury or negating the association. Biological variables (injury-related factors, medical co-morbidities, medications, family history of psychiatric illness, past personal psychiatric history, ongoing substance abuse), the psychosocial network (relationships with family/friends, presence or absence of social support/employment/finances, losses incurred) and cultural factors (value of spirituality, religion, symptom attribution) should all be considered in the formulation.

A comprehensive formulation is important, as it can guide management. It is important to note that, despite the high frequency of concussion in the community, there are less empirical data to guide psychiatric treatment after single or repetitive concussions. As mentioned above, DSM-5 diagnosis of ‘Neurocognitive Disorder due to TBI’ best captures the neuropsychiatric sequelae of TBI as it includes the definition of TBI, its severity, presence/absence of behavioral disturbances and the recovery course. The ICoMP formed to help promote better recovery after concussion recommend early education and providing positive reassurance after concussion. They suggest addressing psychosocial factors (e.g., poor expectations of recovery, negative injury perceptions) and medical co-morbidities (e.g., somatic pain) and avoiding excessive diagnostic testing [22].

### Management

Per our clinical experience, management of NPS after concussion should be multipronged and include a combination of psychotherapy and pharmacotherapy. Appropriate psychiatric treatment should be provided if there is evidence of a mood, anxiety, cognitive or substance use disorder. In people with substance use disorders, referral to outpatient or inpatient substance abuse programs should be considered. It is important to let patients know that both premature return to physical activities and prolonged rest can be harmful. People with persistence of symptoms should be considered for an outpatient neurorehabilitation program [7,56].

Psychotherapy treatments include psychoeducation regarding the natural course of illness, supportive therapy (e.g., providing hope, grief counseling, minimize/remove misattributions associated with brain injury), behavioral therapy (e.g., working on establishing structure during the day, maintaining sleep hygiene, balancing relaxation versus exercise, learning to control impulsivity, anger management) and restorative and compensatory training. The latter is best done in an appropriate neurorehabilitation facility and by working with a team including a neuropsychologist, physical medicine and rehabilitation specialist, occupational therapist, speech-language specialist or physical therapist, depending on the nature of deficits. As symptoms improve, working with vocational therapists is also recommended. The overarching goal should be to minimize disability and improve functionality and productivity.

A systematic review by Cooper *et al*. [57] on persistent postconcussive symptoms after concussion concludes that while therapeutic interventions such as cognitive rehabilitation, psychotherapy and medical/psychiatric integrated treatment of co-morbid medical conditions are promising, there are very few randomized trials and more research including comparative studies are needed to determine the effectiveness of these approaches.

Mindfulness training has been popular and successful in our experience. In addition, Azulay *et al*. conducted a 10-week study to evaluate the effectiveness of the mindfulness-based stress reduction therapy in 22 individuals with concussion. Compared with pre-treatment, they noted post-treatment improvements on measures of quality of life, perceived self-efficacy and cognition including tests of working memory and attention. The improvement in these skills can allow patients to experience personal growth after the emotional trauma of brain injury, and decrease ‘catastrophic symptoms’ that can perpetuate disability [58].

Leddy *et al*. [59] have proposed that whole body aerobic exercise rehabilitation of progressively increasing intensity performed at submaximal level can improve central regulatory and autoregulatory function and help resolve some postconcussive symptoms, especially in athletes. These researchers have developed the Buffalo Concussion Treadmill Test to systematically diagnose postconcussion syndrome, differentiate concussion from other diagnoses (e.g., cervical injury, depression and migraines) and quantify the clinical severity and exercise capacity of concussed patients. The test is based on the Balke cardiac protocol, which imparts a gradual increase in workload. Using this test, they also developed individualized ‘subthreshold exercise treatment programs’ to restore physiology and enhance recovery. Leddy *et al*. [60] propose that return of normal exercise tolerance can be considered an objective physiological biomarker of recovery. With respect to pharmacotherapy, there are some basic principles that need to be followed in the treatment of people with brain injury [61,62]. Pertinent ones include: ‘start low, go slow, but go.’ It is important to start medications at a low dosage, as people with brain injury may be particularly sensitive to them. However, it may be occasionally necessary to utilize high doses of psychotropics in a manner similar to that of idiopathic psychiatric disease. Close regular monitoring for side effects is important and necessary; if possible, avoid medications that are known for lowering seizure threshold (e.g., some tricyclic antidepressants, bupropion), causing weight gain or sedation (tricyclic antidepressants), or have risk of causing tolerance and dependence (e.g., benzodiazepines and opiates); monitor use of ‘as needed’ medicine (i.e., PRN medicines); avoid chasing your tail – for example, using a stimulant followed by a benzodiazepine for treatment of sedation and later increasing the dose of the stimulant to counteract the sedation; practice ‘trial off’ treatment. If not sure about the effectiveness of a medicine, consider tapering and discontinuation and see how the person does off the medicine.

There are no US FDA approved medications for the treatment of majority of NPS associated with concussion and physicians should be mindful to use any pharmacotherapies for the treatment as it is considered ‘off-label’. Leading researchers in the field of brain injury have reviewed the literature on pharmacological treatment for TBI-related neuropsychiatric problems and provided evidence-based guidelines and options for physicians (Neurobehavioral Workgroup, 2006) [63]. They have concluded that in order to establish definitive treatment standards for the TBI population, there is a need for well-designed randomized controlled trials.

There are a few recent review articles on the management of neuropsychiatric aspects of concussions [4,64] and all severities of TBI [65] and the readers are encouraged to review these [66]. Based on our clinical experience, we have also included a table of commonly used medications to treat NPS (Table 1).

**Table 1.** **Psychotropics in the treatment of traumatic brain injury neuropsychiatric symptoms.**

**Psychotropics**	**Target symptoms**
Antidepressants (e.g., SSRIs such as sertraline 25–150 mg, escitalopram 5–20 mg per day; SNRIs such as venlafaxine extended release 37.5–225 mg; duloxetine 30–90 mg)	Depression, impulsivity, anxiety, aggression/agitation

Mood stabilizers (e.g., valproate 125–1000 mg, carbamazepine 100–600 mg per day)	Mood cycling

Atypical antipsychotics (e.g., quetiapine 25–200 mg, risperidone 0.5–4 mg per day)	Psychosis, aggression, agitation, mood cycling, irritability

Dopamine agonists (e.g., methylphenidate 5–20 mg, amantadine 100–300 mg per day)	Inattention, mental fatigue

Cholinesterase inhibitors (e.g., donepezil 5–10 mg per day)	Episodic memory loss

Buspirone (e.g., 10–60 mg per day)	Anxiety, irritability

Beta blockers (e.g., propranolol 30–120 mg per day): – Monitor blood pressure	Aggression

SNRI: Serotonin norepinephrine reuptake inhibitor; SSRI: Selective serotonin reuptake inhibitor.

In summary, despite many advances, there is minimal evidence from randomized controlled trials to guide the treatment and prevention of neuropsychiatric consequences of concussions. In an excellent review titled, “Effort, exaggeration and malingering after concussion,” Silver [67] notes that these symptoms are ‘complex and multi-determined behaviors’ and have a long differential diagnosis which all clinicians should investigate and address to promote healthy recovery. He proposes the following interventions to minimize persistence of neuropsychiatric symptoms: increase expectations of positive recovery; treat depression and anxiety; minimize the stereotype threat that brain injury is associated with cognitive deficits or development of physical/cognitive/emotional symptoms; work on feelings of anger and revenge associated with sustaining a TBI/concussion; address loss aversion – that is, feelings of economic, social and/or functional loss and inability to regain the original self; bear in mind the impact of financial incentives on behavior.

There is clearly a need to conduct large, appropriately controlled management and prevention studies that have statistical power to compare different modalities and to control for a variety of confounding variables. It is also important to also evaluate alternative systems of healthcare delivery, such as telephone- and web-based technologies [68].

## Conclusion

There are many questions that remain unanswered regarding concussions and their chronic neuropsychiatric sequelae. Pertinent questions include: is there a threshold in the number of injuries or severity of injuries for the development of neuropsychiatric sequelae? What are the roles of genetic factors, medical co-morbidities and substance use? Are there early biomarkers that can identify at-risk patients? What are the best clinical diagnostic criteria for CTE? Why do some develop CTE and others do not? Are there any biomarkers – clinical, blood, neuroimaging?

It is imperative that we understand the short-term and long-term sequelae of concussion in order to develop public health policies, create best practice guidelines for return-to-play or return-to-work, and hopefully deploy early therapeutic interventions in the future. However, while we are waiting for the results of well-done scientific studies, it is important to practice good clinical medicine, educate our patients and their family members about taking the necessary steps to avoid future head injuries, conduct comprehensive neuropsychiatric evaluations when symptoms persist, focus on treating treatable symptoms, and not jump to conclusions about causes and associations.

## Future perspective

Even though there has been much focus on concussion in the past decade, particularly given the increasing incidence and intensity of sports and combat-related injuries, the scientific evidence for links between concussion (particularly repetitive concussions) and chronic neurodegenerative sequelae is still limited. Major obstacles in the literature on concussion neuropsychiatry include lack of clinical criteria for CTE, poorly designed studies, and absence of longitudinal follow-up. It is important not to jump to conclusions from case reports and small case-series studies. The time is now ripe for identifying clinical, serum and/or neuroimaging biomarkers by conducting large-scale prospective longitudinal clinicopathological studies to better understand the relationship between concussions and their neuropsychiatric sequelae.

**Executive summary**

**Acute neuropsychiatric symptoms/syndromes**

- Post-concussion syndrome which includes a myriad of emotional, cognitive and behavioral symptoms noted in a majority of people after concussion. Symptoms gradually resolve within a few months.
- Second Impact Syndrome. A rare fatal condition noted after sustaining a second concussion before symptoms from the first have resolved.

**Second impact syndrome**

- A rare fatal condition noted after sustaining a second concussion before symptoms from the first have resolved.

**Chronic neuropsychiatric symptoms/syndromes**

- Emotional and behavioral problems such as mood disturbances, sleep problems and substance abuse.
- Cognitive deficits predominantly involving the domains of executive functioning and memory.
- Chronic traumatic encephalopathy postulated to be a latent neurodegenerative condition in people with multiple concussions. Well-controlled epidemiological studies are needed to validate this diagnosis.

**Evaluation and management**

- Comprehensive neuropsychiatric examination is the current gold standard for diagnosis. Management should be multipronged.
